# 2,5-Dihexyl-3,6-diphenyl­pyrrolo­[3,4-*c*]pyrrole-1,4(2*H*,5*H*)-dione

**DOI:** 10.1107/S1600536810020398

**Published:** 2010-06-05

**Authors:** Resul Sevinçek, Seçil Çelik, Muhittin Aygün, Serap Alp, Şamil Işık

**Affiliations:** a Department of Physics, Dokuz Eylül University, Tınaztepe 35160, Buca-Izmir, Turkey; b Department of Chemistry, Dokuz Eylül University, Tınaztepe 35160, Buca-Izmir, Turkey; c Department of Physics, Ondokuz Mayıs University, Kurupelit-Samsun, Turkey

## Abstract

The asymmetric unit of the title compound, C_30_H_36_N_2_O_2_, contains one half-mol­ecule, the other half being generated by a crystallographic inversion centre. The crystal structure is devoid of any classical hydrogen bonds however, non-classical C—H⋯O inter­actions link the mol­ecules into chains propagating in [001] and a C—H⋯π inter­action leads to the formation of a two-dimensional network in (011).

## Related literature

For the use of diketodiphenyl­pyrrolo­pyrroles as pigmants, see: Iqbal *et al.* (1988 ▶); Herbst & Hunger (1993 ▶). For related structures, see; Hirota *et al.* (2006 ▶); Mizuguchi (1998 ▶). For the synthesis of the starting reactant, see: Morton *et al.* (2002 ▶).
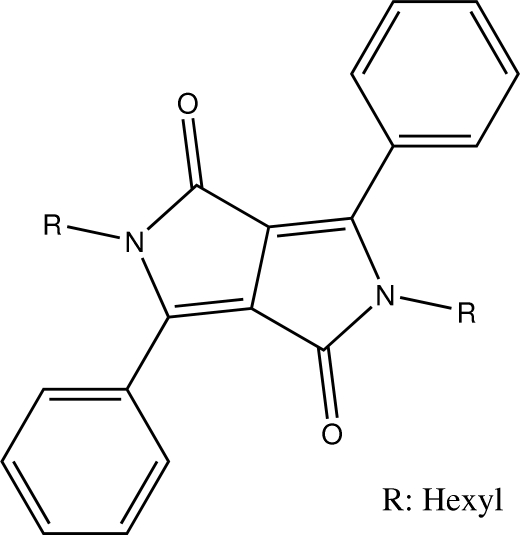


## Experimental

### 

#### Crystal data

C_30_H_36_N_2_O_2_
*M* *_r_* = 456.61
Monoclinic, 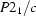

*a* = 13.4809 (11) Å
*b* = 5.5393 (3) Å
*c* = 17.4838 (14) Å
β = 90.218 (7)°
*V* = 1305.59 (17) Å^3^
*Z* = 2
Mo *K*α radiation
μ = 0.07 mm^−1^
*T* = 293 K
0.48 × 0.22 × 0.07 mm

#### Data collection

Stoe IPDS 2 diffractometer
Absorption correction: integration (North *et al.*, 1968 ▶) *T*_min_ = 0.963, *T*_max_ = 0.989
9230 measured reflections
2989 independent reflections
1604 reflections with *I* > 2σ(*I*)
*R*_int_ = 0.038

#### Refinement

*R*[*F* ^2^ > 2σ(*F* ^2^)] = 0.048
*wR*(*F* ^2^) = 0.136
*S* = 0.86
2989 reflections
172 parameters
H atoms treated by a mixture of independent and constrained refinement
Δρ_max_ = 0.13 e Å^−3^
Δρ_min_ = −0.12 e Å^−3^

### 

Data collection: *X-AREA* (Stoe & Cie, 2002 ▶); cell refinement: *X-AREA*; data reduction: *X-RED32* (Stoe & Cie, 2002 ▶); program(s) used to solve structure: *SHELXS97* (Sheldrick, 2008 ▶); program(s) used to refine structure: *SHELXL97* (Sheldrick, 2008 ▶); molecular graphics: *PLUTON* (Spek, 2009 ▶); software used to prepare material for publication: *WinGX* (Farrugia, 1999 ▶).

## Supplementary Material

Crystal structure: contains datablocks I, global. DOI: 10.1107/S1600536810020398/su2177sup1.cif

Structure factors: contains datablocks I. DOI: 10.1107/S1600536810020398/su2177Isup2.hkl

Additional supplementary materials:  crystallographic information; 3D view; checkCIF report

## Figures and Tables

**Table 1:** Hydrogen-bond geometry (Å, °)

*D*—H⋯*A*	*D*—H	H⋯*A*	*D*⋯*A*	*D*—H⋯*A*
C1—H1⋯O2^i^	0.93 (2)	2.57 (2)	3.226 (2)	127.8 (17)
C3—H3⋯O2^ii^	0.98 (2)	2.43 (2)	3.316 (3)	149.6 (18)

Symmetry codes: (i) 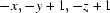; (ii) 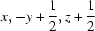.

**Fig. 1.**
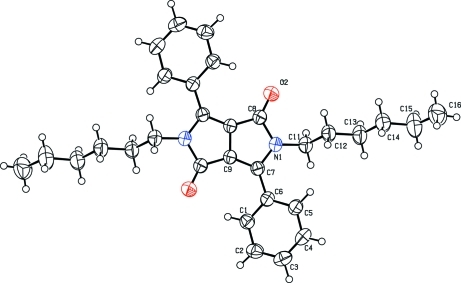
A view of the molecular structure of the title compound, with displacement ellipsoids drawn at the 50% probability level. The unlabelled atoms are related by the inversion center (symmetry code = -x, 1-y, 1-z).

**Fig. 2.**
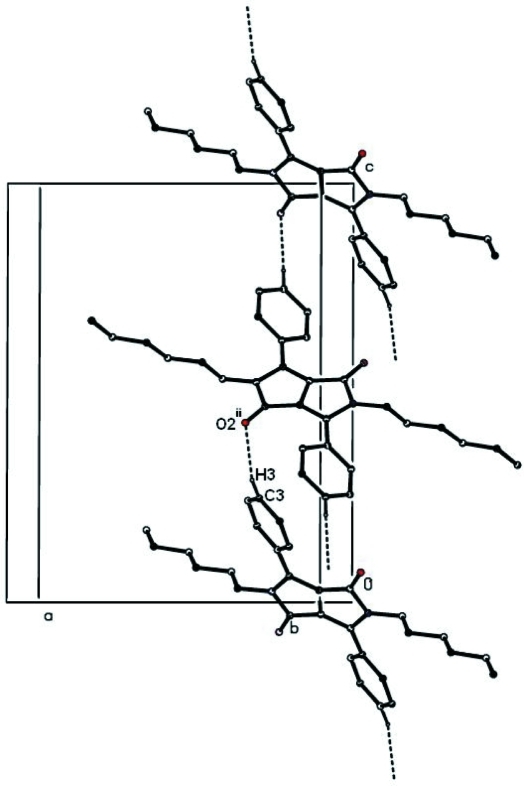
A view of the crystal packing of the title compound, illustrating the formation of the C-H···O hydrogen bonded chain propagating along the c-axis (H-atoms not involved in this interaction have been omited for clarity; Symmetry code: (ii) x,1/2-y,1/2+z).

**Fig. 3.**
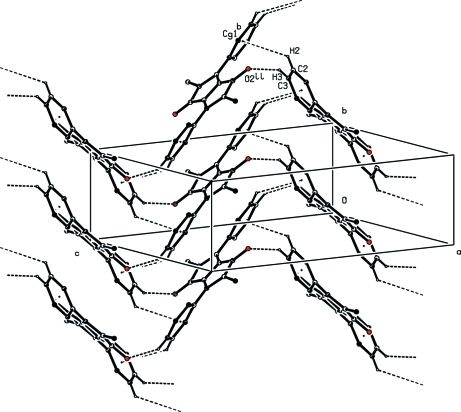
A view of the crystal packing of the title compound, illustrating the C–H ··· O and C – H ··· π interactions which contributes to the formation of a two-dimensional network in (011). (hexyl groups and H-atoms not involved in this interaction have been omited for clarity; Symmetry codes: (ii) x,1/2-y,1/2+z, (b) -x,1/2+y,1/2-z).

## supplementary crystallographic information

### Comment

Diketodiphenylpyrrolopyrroles are industrially important red pigments (Herbst & Hunger, 1993). The success of these compounds as pigments relies, in part, on their high light fastness and very low solubility in most common solvents. This state of low solubility is presumed to result from the presence of a 2-dimensional network formed by intermolecular hydrogen bonds (C-H···O), combined with π—π and Van der Waals interactions of the aryl substituents between layers of molecules (Iqbal *et al.*, 1988).

The molecular structure of the title molecule is illustrated in Fig. 1. It is situated on an inversion center. The pyrrolopyrrole 8-membered ring is almost planar (C8 has a maximum deviation of 0.4636 (15) Å). Because of the steric effect of the alkyl group, the pyrrolopyrrole and phenyl rings are not coplanar. The dihedral angle between the mean planes of these rings is 34.38 (9) °.

In the crystal molecules are linked into one dimensional chains, generated by two–folded screw operation along the c-axis of the unit cell, via weak C3-H3 ···O2^ii^ interactions (Symmetry code: (ii) x,1/2-y, 1/2 +z]; Fig. 2 and Table 1]. There is also a C–H ··· π interaction in the crystal structure involving the phenyl ring (C1-C6; centroid Cg) and atom C2 in a neighbouring molecule [C2—H2 ··· Cg^b^: H2···Cg^b^ = 2.90Å and C2—H2···Cg^b^ = 129°; symmetry code: (b) - x, -1/2 + y, 3/2 - z]. This interaction forms a one dimensional chain running along the c-axis. These two interactions lead to the formation of a two-dimensional network in (011) [Fig.  3].

### Experimental

The starting reactant, 3,6-diphenyl-2,5-dihydro-1,4-diketopyrrolo[3,4-c]pyrrole-1,4-dione (L), was prepared following the literature procedure (Morton *et al.*, 2002). The synthesis of the title compound was carried out under a nitrogen atmosphere. L (0.59 g, 0.00204 mol) was stirred in 1-methyl-2-pyrolidinone (30 mL) at room temperature. Potassium-tert-butoxide (0.230 g, 0.00816 mmol) was then added follwed by the addition of the n-hexyl bromide (0.340 g, 0.00204 mmol) and the mixture was stirred for 18 hours, after which it was poured into 30 mL of cold water. The precipitate formed was filtered off and the crude product purified by column chromatography, using ethyl acetate/n-hexane (1:3) as eluent. Re-crystallization from methanol produced orange prism-like crystals of the title compound, suitable for X-ray analysis. Yield: 44%; m.p. = 243°C; IR (KBr): ν_=C—H (ger.)_ 3055, ν_-C—H (ger.)_ 2847-2911, ν_-C=O (ger.)_ 1674; ^1^H NMR (CDCl3) : δ (ppm) 3.74 (t, 2H); 1.59 (p, 2H); 1.24 (6*H*, m); 0.82 (t, 3H)

### Refinement

Aromatic H-atoms were located in difference Fourier maps and freeely refined. The others H-atoms were included in calculated positions and treated as riding atoms: C–H = 0.96 Å for methyl H-atoms and 0.97 Å for methylene H atoms, with U_iso_(H) = k × U_eq_(C), where k = 1.5 for methyl H-atosm and = 1.2 for methylene H-atoms.

### Crystal data

**Table:**

C_30_H_36_N_2_O_2_	*F*(000) = 492
*M**_r_* = 456.61	*D*_x_ = 1.161 Mg m^−^^3^
Monoclinic, *P*2_1_/*c*	Mo *K*α radiation, λ = 0.71073 Å
Hall symbol: -P 2ybc	Cell parameters from 6690 reflections
*a* = 13.4809 (11) Å	θ = 1.5–29.6°
*b* = 5.5393 (3) Å	µ = 0.07 mm^−^^1^
*c* = 17.4838 (14) Å	*T* = 293 K
β = 90.218 (7)°	Prism, orange
*V* = 1305.59 (17) Å^3^	0.48 × 0.22 × 0.07 mm
*Z* = 2	

### Data collection

**Table:**

Stoe IPDS 2 diffractometer	2989 independent reflections
Radiation source: fine-focus sealed tube	1604 reflections with *I* > 2σ(*I*)
graphite	*R*_int_ = 0.038
Detector resolution: 6.67 pixels mm^-1^	θ_max_ = 27.5°, θ_min_ = 1.5°
ω and φ scans	*h* = −17→17
Absorption correction: integration (North *et al.*, 1968)	*k* = −7→6
*T*_min_ = 0.963, *T*_max_ = 0.989	*l* = −22→19
9230 measured reflections	

### Refinement

**Table:**

Refinement on *F*^2^	Primary atom site location: structure-invariant direct methods
Least-squares matrix: full	Secondary atom site location: difference Fourier map
*R*[*F*^2^ > 2σ(*F*^2^)] = 0.048	Hydrogen site location: inferred from neighbouring sites
*wR*(*F*^2^) = 0.136	H atoms treated by a mixture of independent and constrained refinement
*S* = 0.86	*w* = 1/[σ^2^(*F*_o_^2^) + (0.0797*P*)^2^] where *P* = (*F*_o_^2^ + 2*F*_c_^2^)/3
2989 reflections	(Δ/σ)_max_ < 0.001
172 parameters	Δρ_max_ = 0.13 e Å^−^^3^
0 restraints	Δρ_min_ = −0.12 e Å^−^^3^

### Special details

**Table:**

Geometry. All esds (except the esd in the dihedral angle between two l.s. planes) are estimated using the full covariance matrix. The cell esds are taken into account individually in the estimation of esds in distances, angles and torsion angles; correlations between esds in cell parameters are only used when they are defined by crystal symmetry. An approximate (isotropic) treatment of cell esds is used for estimating esds involving l.s. planes.
Refinement. Refinement of *F*^2^ against ALL reflections. The weighted *R*-factor wR and goodness of fit *S* are based on *F*^2^, conventional *R*-factors *R* are based on *F*, with *F* set to zero for negative *F*^2^. The threshold expression of *F*^2^ > σ(*F*^2^) is used only for calculating *R*-factors(gt) etc. and is not relevant to the choice of reflections for refinement. *R*-factors based on *F*^2^ are statistically about twice as large as those based on *F*, and *R*- factors based on ALL data will be even larger.

### Fractional atomic coordinates and isotropic or equivalent isotropic displacement parameters (Å^2^)

**Table:**

	*x*	*y*	*z*	*U*_iso_*/*U*_eq_	
H1	−0.0038 (15)	−0.043 (4)	0.5980 (12)	0.059 (6)*	
H3	0.1434 (16)	−0.228 (4)	0.7917 (14)	0.081 (7)*	
H5	0.2139 (14)	0.396 (4)	0.6811 (11)	0.060 (6)*	
H2	0.0192 (15)	−0.307 (4)	0.6947 (12)	0.066 (6)*	
H4	0.2334 (15)	0.128 (4)	0.7809 (13)	0.069 (6)*	
O2	0.15927 (10)	0.7876 (3)	0.42882 (8)	0.0616 (4)	
N1	0.15436 (10)	0.5041 (3)	0.52594 (8)	0.0455 (4)	
C1	0.04698 (14)	−0.0115 (3)	0.63284 (11)	0.0481 (5)	
C2	0.06082 (15)	−0.1683 (4)	0.69339 (12)	0.0550 (5)	
C3	0.13127 (16)	−0.1198 (4)	0.74799 (13)	0.0593 (5)	
C4	0.18816 (16)	0.0862 (4)	0.74268 (12)	0.0596 (6)	
C5	0.17535 (14)	0.2435 (4)	0.68207 (11)	0.0525 (5)	
C6	0.10474 (12)	0.1964 (3)	0.62547 (10)	0.0435 (4)	
C7	0.08382 (12)	0.3606 (3)	0.56225 (10)	0.0425 (4)	
C8	0.11058 (13)	0.6500 (4)	0.46795 (10)	0.0470 (5)	
C9	−0.00613 (12)	0.4115 (3)	0.52888 (10)	0.0441 (4)	
C11	0.26221 (12)	0.4958 (4)	0.53304 (12)	0.0500 (5)	
H11A	0.2909	0.4832	0.4824	0.060*	
H11B	0.2808	0.3524	0.5615	0.060*	
C12	0.30505 (12)	0.7163 (4)	0.57300 (12)	0.0540 (5)	
H12A	0.2889	0.8593	0.5434	0.065*	
H12B	0.2745	0.7328	0.6229	0.065*	
C13	0.41624 (14)	0.7009 (4)	0.58286 (14)	0.0643 (6)	
H13A	0.4318	0.5604	0.6138	0.077*	
H13B	0.4462	0.6777	0.5331	0.077*	
C14	0.46184 (14)	0.9206 (4)	0.61985 (14)	0.0679 (6)	
H14A	0.4297	0.9475	0.6687	0.081*	
H14B	0.4482	1.0598	0.5878	0.081*	
C15	0.57231 (17)	0.9048 (5)	0.63300 (19)	0.0942 (9)	
H15A	0.5860	0.7609	0.6628	0.113*	
H15B	0.6045	0.8853	0.5839	0.113*	
C16	0.6173 (2)	1.1131 (5)	0.67233 (17)	0.0936 (6)	
H16A	0.6873	1.0866	0.6783	0.140*	
H16B	0.5876	1.1322	0.7218	0.140*	
H16C	0.6065	1.2565	0.6426	0.140*	

### Atomic displacement parameters (Å^2^)

**Table:**

	*U*^11^	*U*^22^	*U*^33^	*U*^12^	*U*^13^	*U*^23^
O2	0.0544 (8)	0.0736 (10)	0.0566 (8)	−0.0152 (7)	−0.0035 (6)	0.0179 (8)
N1	0.0406 (7)	0.0486 (9)	0.0472 (8)	−0.0032 (7)	−0.0068 (6)	0.0032 (7)
C1	0.0490 (10)	0.0452 (11)	0.0500 (11)	−0.0005 (8)	−0.0029 (8)	−0.0015 (9)
C2	0.0586 (12)	0.0448 (12)	0.0615 (13)	0.0035 (9)	0.0062 (9)	0.0052 (10)
C3	0.0647 (12)	0.0595 (13)	0.0538 (12)	0.0145 (10)	0.0018 (10)	0.0119 (11)
C4	0.0600 (12)	0.0686 (14)	0.0502 (12)	0.0080 (10)	−0.0142 (10)	0.0043 (11)
C5	0.0522 (10)	0.0499 (12)	0.0552 (12)	0.0003 (9)	−0.0125 (9)	0.0026 (10)
C6	0.0444 (9)	0.0416 (10)	0.0445 (10)	0.0029 (7)	−0.0051 (7)	−0.0026 (8)
C7	0.0476 (9)	0.0385 (10)	0.0412 (9)	−0.0037 (7)	−0.0069 (7)	−0.0028 (8)
C8	0.0474 (9)	0.0509 (11)	0.0427 (10)	−0.0061 (8)	−0.0057 (8)	0.0024 (9)
C9	0.0456 (9)	0.0460 (10)	0.0407 (9)	−0.0070 (8)	−0.0073 (7)	0.0019 (8)
C11	0.0420 (9)	0.0527 (11)	0.0554 (11)	0.0007 (8)	−0.0030 (8)	−0.0034 (10)
C12	0.0422 (10)	0.0539 (12)	0.0657 (13)	−0.0017 (8)	−0.0043 (8)	−0.0028 (10)
C13	0.0442 (10)	0.0659 (14)	0.0827 (15)	−0.0004 (9)	−0.0086 (10)	−0.0067 (12)
C14	0.0519 (11)	0.0711 (14)	0.0806 (15)	−0.0086 (10)	−0.0072 (10)	−0.0043 (13)
C15	0.0580 (13)	0.094 (2)	0.131 (2)	−0.0089 (13)	−0.0183 (14)	−0.0201 (19)
C16	0.0949 (17)	0.092	0.094	−0.0241 (15)	−0.0145 (15)	−0.0125 (17)

### Geometric parameters (Å, °)

**Table:**

O2—C8	1.218 (2)	C9—C8^i^	1.450 (2)
N1—C7	1.394 (2)	C11—C12	1.520 (3)
N1—C8	1.423 (2)	C11—H11A	0.9700
N1—C11	1.459 (2)	C11—H11B	0.9700
C1—C2	1.382 (3)	C12—C13	1.511 (3)
C1—C6	1.396 (3)	C12—H12A	0.9700
C1—H1	0.93 (2)	C12—H12B	0.9700
C2—C3	1.371 (3)	C13—C14	1.508 (3)
C2—H2	0.95 (2)	C13—H13A	0.9700
C3—C4	1.378 (3)	C13—H13B	0.9700
C3—H3	0.99 (2)	C14—C15	1.509 (3)
C4—C5	1.382 (3)	C14—H14A	0.9700
C4—H4	0.93 (2)	C14—H14B	0.9700
C5—C6	1.395 (3)	C15—C16	1.473 (3)
C5—H5	0.99 (2)	C15—H15A	0.9700
C6—C7	1.458 (2)	C15—H15B	0.9700
C7—C9	1.373 (2)	C16—H16A	0.9600
C8—C9^i^	1.450 (2)	C16—H16B	0.9600
C9—C9^i^	1.418 (4)	C16—H16C	0.9600
			
C7—N1—C8	111.50 (14)	N1—C11—H11B	109.0
C7—N1—C11	128.63 (15)	C12—C11—H11B	109.0
C8—N1—C11	119.27 (15)	H11A—C11—H11B	107.8
C2—C1—C6	121.04 (19)	C13—C12—C11	112.49 (16)
C2—C1—H1	118.7 (13)	C13—C12—H12A	109.1
C6—C1—H1	120.2 (13)	C11—C12—H12A	109.1
C3—C2—C1	120.1 (2)	C13—C12—H12B	109.1
C3—C2—H2	123.2 (13)	C11—C12—H12B	109.1
C1—C2—H2	116.7 (13)	H12A—C12—H12B	107.8
C2—C3—C4	120.0 (2)	C14—C13—C12	113.97 (18)
C2—C3—H3	122.2 (14)	C14—C13—H13A	108.8
C4—C3—H3	117.8 (14)	C12—C13—H13A	108.8
C3—C4—C5	120.37 (19)	C14—C13—H13B	108.8
C3—C4—H4	121.3 (14)	C12—C13—H13B	108.8
C5—C4—H4	118.2 (14)	H13A—C13—H13B	107.7
C4—C5—C6	120.6 (2)	C13—C14—C15	114.8 (2)
C4—C5—H5	119.1 (12)	C13—C14—H14A	108.6
C6—C5—H5	120.1 (12)	C15—C14—H14A	108.6
C5—C6—C1	117.88 (17)	C13—C14—H14B	108.6
C5—C6—C7	123.43 (17)	C15—C14—H14B	108.6
C1—C6—C7	118.55 (16)	H14A—C14—H14B	107.5
C9—C7—N1	106.98 (15)	C16—C15—C14	115.5 (2)
C9—C7—C6	128.20 (16)	C16—C15—H15A	108.4
N1—C7—C6	124.79 (15)	C14—C15—H15A	108.4
O2—C8—N1	122.19 (16)	C16—C15—H15B	108.4
O2—C8—C9^i^	133.95 (17)	C14—C15—H15B	108.4
N1—C8—C9^i^	103.87 (15)	H15A—C15—H15B	107.5
C7—C9—C9^i^	109.86 (18)	C15—C16—H16A	109.5
C7—C9—C8^i^	142.35 (17)	C15—C16—H16B	109.5
C9^i^—C9—C8^i^	107.79 (18)	H16A—C16—H16B	109.5
N1—C11—C12	113.00 (15)	C15—C16—H16C	109.5
N1—C11—H11A	109.0	H16A—C16—H16C	109.5
C12—C11—H11A	109.0	H16B—C16—H16C	109.5
			
C6—C1—C2—C3	0.8 (3)	C1—C6—C7—N1	−149.42 (17)
C1—C2—C3—C4	0.3 (3)	C7—N1—C8—O2	−179.45 (18)
C2—C3—C4—C5	−0.7 (3)	C11—N1—C8—O2	−7.6 (3)
C3—C4—C5—C6	0.1 (3)	C7—N1—C8—C9^i^	0.6 (2)
C4—C5—C6—C1	0.9 (3)	C11—N1—C8—C9^i^	172.40 (15)
C4—C5—C6—C7	176.70 (18)	N1—C7—C9—C9^i^	−0.2 (2)
C2—C1—C6—C5	−1.4 (3)	C6—C7—C9—C9^i^	177.71 (19)
C2—C1—C6—C7	−177.34 (18)	N1—C7—C9—C8^i^	−179.4 (2)
C8—N1—C7—C9	−0.3 (2)	C6—C7—C9—C8^i^	−1.5 (4)
C11—N1—C7—C9	−171.14 (17)	C7—N1—C11—C12	−111.5 (2)
C8—N1—C7—C6	−178.23 (17)	C8—N1—C11—C12	78.2 (2)
C11—N1—C7—C6	10.9 (3)	N1—C11—C12—C13	177.67 (17)
C5—C6—C7—C9	−142.7 (2)	C11—C12—C13—C14	177.97 (19)
C1—C6—C7—C9	33.0 (3)	C12—C13—C14—C15	177.7 (2)
C5—C6—C7—N1	34.9 (3)	C13—C14—C15—C16	−177.3 (3)

Symmetry codes: (i) −*x*, −*y*+1, −*z*+1.

### Hydrogen-bond geometry (Å, °)

**Table:**

*D*—H···*A*	*D*—H	H···*A*	*D*···*A*	*D*—H···*A*
C1—H1···O2^i^	0.93 (2)	2.57 (2)	3.226 (2)	127.8 (17)
C3—H3···O2^ii^	0.98 (2)	2.43 (2)	3.316 (3)	149.6 (18)

Symmetry codes: (i) −*x*, −*y*+1, −*z*+1; (ii) *x*, −*y*+1/2, *z*+1/2.
